# Engagement in Stroke Prevention Shared Decision Making in Older Adults with Atrial Fibrillation and Multimorbidity

**DOI:** 10.1093/geroni/igaf122.3337

**Published:** 2025-12-31

**Authors:** Hawa Abu, Jane Saczynski, Jerry Gurwitz, Kathleen Mazor, Robert Goldberg, David Dosa, Alok Kapoor, David McManus

**Affiliations:** University of Massachusetts Chan Medical School, Worcester, Massachusetts, United States; Northeastern University, Boston, Massachusetts, United States; University of Massachusetts Chan Medical School, Worcester, Massachusetts, United States; University of Massachusetts Chan Medical School, Worcester, Massachusetts, United States; University of Massachusetts Chan Medical School, Worcester, Massachusetts, United States; University of Massachusetts Chan Medical School, Worcester, Massachusetts, United States; University of Massachusetts Chan Medical School, Worcester, Massachusetts, United States; University of Massachusetts Chan Medical School, Worcester, Massachusetts, United States

## Abstract

**Background:**

Older adults with atrial fibrillation (AF) and multimorbidity are posed with stroke prevention decisional conflicts. Understanding the factors that predict engagement in shared decision making (SDM) can enhance patient-centered care.

**Objective:**

To examine the extent of engagement in SDM on stroke prevention among older adults with AF and multimorbidity.

**Methods:**

A prospective cohort study (2016-2018) enrolled patients aged ≥65 years with AF from clinics in Massachusetts and Georgia. Those with one or more chronic conditions were included in the present study. At one-year follow-up, participants on oral anticoagulation were asked about their involvement in both choosing to be on anticoagulation and selecting an anticoagulant. Engagement in SDM was categorized as fully engaged (affirmative to both questions), partially engaged (affirmative to one), or not engaged (non-affirmative to both).

**Results:**

Among 808 participants (mean age 75 years, 48% women, 88% White), 33% were engaged in both aspects of SDM, 28% participated in anticoagulation initiation, 4% in selecting a specific anticoagulant, and 35% did not actively participate in either. Non-engaged patients were more likely to be older, non-White, have lower education levels, cognitive impairment, frailty, poor self-rated health, greater dependency in daily activities, higher comorbidity burden, and lower perceived benefit from anticoagulation.

**Conclusions:**

In managing older adults with AF and multimorbidity, healthcare providers should be aware of vulnerable populations that are at risk of being excluded from proper engagement in SDM for stroke prevention to ensure a more tailored approach to providing holistic and patient-centered care for improved outcomes.

